# A review: effects of neurofeedback on patients with mild cognitive impairment (MCI), and Alzheimer’s disease (AD)

**DOI:** 10.3389/fnhum.2023.1331436

**Published:** 2024-02-14

**Authors:** Miyako Tazaki

**Affiliations:** Department of Psychology, Faculty of Medicine, Toho University, Tokyo, Japan

**Keywords:** neurofeedback, cognitive decline, mild cognitive impairment, Alzheimer’s disease, dementia

## Abstract

Neurofeedback training (NFT) is a non-invasive method and has been shown to be effective for attention deficit/hyperactivity disorder (ADHD) and various psychiatric disorders. The aim of this paper is to evaluate the effectiveness of NFT for patients with Mild Cognitive Impairment (MCI) and Alzheimer’s disease (AD) or Vascular Diseases (VD), so that we searched research articles from four databases, using the keywords neurofeedback, elderly, MCI, AD, VD, and dementia. As a result, 13 articles were identified regarding the effectiveness of NFT in patients with MCI and AD. Although each study differed in study design, training protocol, electroencephalogram (EEG) electrode placement, and reward and inhibition frequency bands, all were shown to enhance memory, attention, and other cognitive abilities. Additional well-designed, randomized studies with sufficient power are needed to further confirm the effectiveness of NFT.

## Introduction

According to the World Alzheimer Report 2015 (Alzheimer’s Disease International, 2015), the number of patients with dementia worldwide, currently 46 million, is expected to increase to more than 130 million by 2050, although currently available drugs for dementia may be able to reduce core symptoms such as memory disruptions, disorientation, performance disorders, depression, and anxiety, they cannot cure the disease itself. Consequently, it has been announced by the WHO that prevention is the most important target for dementia.

Dementia refers to a group of symptoms associated with the decline of memory or other thinking skills. The symptoms associated with dementia must be severe enough to reduce a person’s ability to perform everyday activities. Alzheimer’s disease (AD) accounts for 60–80% of all cases of dementia. Vascular Dementia (VD) is the second most common type of dementia. Other forms of dementia include frontotemporal dementia and Lewy body dementia (Ubhi and Masliah, 2013). The main symptom of dementia is the deterioration of cognitive function caused by the destruction of neurons in the brain. This deterioration typically manifests as memory loss, confusion about time and place, mood changes, and difficulties concentrating, carrying out familiar daily tasks, following a conversation, or word retrieval (World Health Organization, 2023).

It was recently suggested that EEG plays a special role in the diagnosis of AD. EEG revealed an increased activity in the theta band and decreased activity in the alpha and beta bands of the heathy elderly (Ubhi and Masliah, 2013). In addition, spectral coherence between the two hemispheres decreased between the alpha and beta frequency bands, and the amplitude of the peak of the alpha frequency band reduced (van der Zande et al., 2018). Mild cognitive impairment (MCI) represents a transitional stage between healthy aging and dementia, and it affects 10–15% of the population aged over 65 years. The neurobiological features of MCI are hypoperfusion and hypometabolism in the temporoparietal cortices as well as medial temporal lobe atrophy particularly in the rhinal cortices (Angelakis et al., 2007). In dementia with Lewy bodies, slow and sharp waves generally appear in the subcortical and temporal lobe structures on EEG (Angelakis et al., 2007).

As a preventive measure for dementia, healthy dietary habits (Anderson, 2019), moderate exercise (Swaminathan and Jicha, 2014), smoking cessation (Karstens et al., 2019), sobriety (Ahlskog et al., 2011), adequate sleep (World Health Organization, 2014), and good relationships with people (Ridley et al., 2013) are strongly recommended. Lifestyle diseases (Spira et al., 2014), such as diabetes and high blood pressure, can accelerate arteriosclerosis within the brain, potentially leading to cerebral infarction and hemorrhages, which can cause vascular dementia. It has been reported that AD and vascular dementia are two to four times more common in patients with diabetes. In addition to the above measures, over the last few years, research on the effects of NFT in elderly people with and without dementia has gradually increased.

Neurofeedback training is a type of biofeedback, referred to as EEG biofeedback. Based on operant conditioning paradigm, brainwaves of a participant are measured and analyzed, and spontaneous auditory and visual stimuli are given as the desirable brain activity are rewarded and the undesirable brain activity are inhibited, to regulate the brain for the better. Nowadays, particularly in North American and European countries, NFT has been applied as a non-pharmacological intervention to patients with a wide range of mental disorders, such as attention deficit hyperactivity disorder (ADHD) (Enriquez-Geppert et al., 2019; Van Doren et al., 2019), anxiety (Chen et al., 2021), depression (Fernández-Alvarez et al., 2022), insomnia (Lambert-Beaudet et al., 2021) and epilepsy (Monderer et al., 2002). NFT is also a form of neuromodulation, but simpler and less expensive than other devices used to measure brainwaves, such as EEG, functional magnetic resonance imaging (fMRI), and near-infrared spectroscopy. NFT requires a personal computer, monitors, and an amplifier connected to electrodes, which are placed on the patient’s head and earlobes. The placement of the electrodes is determined by the patient’s arousal level and the cognitive obstacle.

Major NFT training protocols include the beta/SMR and alpha/theta training and iftar-low frequency training. The beta/SMR training is used to train the beta activity, which is associated with conscious precision focus, and the ability to solve problems. Beta/SMR training improves focus and attention and cognitive processing and is usually conducted with the eyes open. Alpha/theta training is typically used for stress reduction and to attain peak performance by enhancing the alpha and theta waves with the eyes closed.

The purpose of this paper was to gather and review current evidence related to NFT to determine whether NFT is effective measure for cognitive impairment in the elderly with MCI and AD or VD.

## Method

We searched for articles within the PubMed, Cochrane Library, Web of Science, and PsycINFO databases using the following keywords on October 15, 2023, (“neurofeedback” or “EEG neurofeedback”) AND (elderly), (neurofeedback OR “EEG neurofeedback”) AND (dementia) OR (mild cognitive impairment) OR (Alzheimer’s disease) OR (Vascular dementia).

## Results

We found seven research articles applied NFT a healthy elderly population, and only six articles that examined the efficacy of NFT in elderly individuals with MCI, and eight articles on AD. No article was found on VD. Research of NFT on healthy elderly showed improvement of cognitive processing speed and executive function (Katsel et al., 2018), attention (Becerra et al., 2012), and working memory (Wang and Hsieh, 2013; Reis et al., 2016), and memory performance (Yamaguchi and Tazaki, 2018), and enhance Quality of Life (Lecomte and Juhel, 2011).

## Effects of EEG NFT on the elderly with MCI, and AD

Six researches on NFT on patients with MCI, and five research on NFT with patients AD or VD and other were found and described in order of the year of publication:

(1) NFT on patients with MCI and dementia

Fotuhi et al. (2016), Van Eijk et al. (2017) trained 127 elderly participants with MCI (with an average age of 70.69 years) during a personalized 12-week “Brain fitness Program.” In the program, each patient received weekly personalized cognitive stimulation, neurofeedback training, and brain coaching/counseling for eating a Mediterranean diet, taking omega-3 supplements, increasing fitness, and practicing mindfulness meditation. The post program testing showed that 84% of the patients experienced statistically significant improvements in their cognitive function, but there were no clear effects on the volume of the hippocampus.

Similarly, Fotuhi et al. (2016), Lavy et al. (2019) conducted another research with 11 MCI patients (mean age: 71.93 years). The participants were divided into two groups; a control group receiving sham NFTs and an experimental group receiving upward NFTs with alpha training and reported improvement in performance in complex memory, cognitive flexibility, composite attention, reaction time and executive functions.

Jang et al. (2019), Lavy et al. (2019) trained 5 MCI patients (mean age:66.6 years) with 16 sessions of NF training (twice a week for 8 weeks) and reported that cognitive function of the patients improved significantly in domains such as composite memory, cognitive flexibility, complex attention, reaction time, and executive function.

Jirayucharoensak et al. (2019) evaluated NFT on the cognitive performance in 54 healthy elderly participants and 65 individuals with anamnestic MCI (mean age; 71.7). All participants received Game-based NFT 20 sessions for 30 min 2–3 sessions per week, and all of them significantly improved spatial WM—a characteristic of amnestic MCI—and rapid visual processing.

Jang et al. (2019), Marlats et al. (2019) published a randomized controlled trial protocol to compare the effect NFT on 20 MCI patients (mean age: 76.1 years). NFT group received SMR upward at CZ 2–3 times per week for 4 months and reported improvement with multiple cognitive tests, compared to the control group in psycho-pedagogical care.

Marlats et al. (2019), Li et al. (2020) trained 40 MCI patients (mean age: 54.3 years) of upward training of alpha, and beta/alpha ratio in 10 sessions. All the patients increased overall connectivity in delta, theta, and beta bands. The results indicate that NFT can improve the brain functional connectivity of people with MCI. Coherence and phase synchronization analysis can objectively and accurately evaluate improvements in functional connectivity.

Li et al. (2020), Trambaiolli et al. (2021) published a systematic review of NFT protocols for dementia and MCI and reported most patients of 10 research (*N* = 81) showed improvement in different cognitive tests applying NFT. The authors pointed out that data from RCT remains scarce, and clinical evidence based on standardized metrics is still inconclusive.

(2) NFT on patients with AD or VD

Luijmes et al. (2016), Trambaiolli et al. (2021) conducted NFT for 10 participants with AD (with an average age of 71.5 years) who were on medication as a pilot study. Post-training of Cambridge Cognitive Examination scores indicated that NFT had a beneficial effect on cognitive functions like information recognition, short-term memory, and learning in patients with AD. Similar results were found in the following two experiments.

Luijmes et al. (2016), Surmeli et al. (2016) assessed the effects of NFT on 20 participants with AD and VD (with an average age of 68.9 years) using qEEG NFT. All patients were on medication and trained in appropriate neurofeedback protocols specific to the individual over different time periods. The results indicated that NFT was effective based on Mini-Mental State Examination scores before and after treatment. On average, scores improved statistically significantly.

Surmeli et al. (2016), Hohenfeld et al. (2017) conducted NFT on 16 healthy elderly subjects (mean age 63.5 years) and 10 AD prodromal patients (mean age 66.2 years). Four additional healthy subjects served as a false-feedback condition to validate the paradigm (WMS backward digit-span) With neurofeedback training, patients with prodromal AD showed improved visuospatial memory performance. The healthy subjects also performed better on a working memory task (WMS backward digit-span), and visuospatial memory performance. Both groups were able to elicit para hippocampal activation during training. Although no significant changes in brain activation were observed, Granger causality analysis revealed changes in brain connectivity during the training period. Controls who received sham feedback showed neither cognitive enhancement nor para hippocampal activation.

Hohenfeld et al. (2017), Galvin-McLaughlin et al. (2022) used Brain Computer Interface (BCI) NFT to improve visual attention and language skills to six mild AD (mean age: 57.6 years) for 4–7 weeks. The study protocol consisted of a within-subject A-B design with multiple data collection sessions both before and during administration of the intervention. In the baseline phase, participants received RSVP Keyboard training without NFT. In the intervention phase, the training incorporated NFB. As the results, increase of frontal power theta with stable beta power was reported indicate feasibility of NFT.

Galvin-McLaughlin et al. (2022), Vilou et al. (2023) reviewed the studies using NFT in rehabilitation of memory deficits in patients with dementia, multiple sclerosis, strokes, and traumatic brain injury. In the review of NFT on dementia, research design and protocol of NFT varied such as SMR/Beta training or Beta or alpha wave enhancement, but addressed that patients showing memory loss and cognitive deficits and behavioral changes altered for the better by NFT.

## Discussion

Neurofeedback training has proven to be very effective in reducing problem behaviors by suppressing abnormally high delta and theta wave amplitudes in ADHD and developmentally disabled children. Similarly, abnormally high theta EEG activity is thought to best predict age-related cognitive dysfunction. Therefore, NFT studies of older adults with MCI or dementia used NFT protocols in which abnormal high wave amplitudes were suppressed by increasing alpha power training or alpha/beta training over 10 to 30 sessions. However, since healthy lifestyle composing of healthy diet with adequate amount of protein intake and carbohydrate restriction, as well as moderate exercise, adequate sleep, and social relationships, have a greater impact on cognitive function in the elderly, it would be more effective to apply NFT to dementia patients after improving these factors. However, research on NFTs for older adults with MCI and dementia only began in 2016, and the number of studies is limited. This may be because the NFT researchers themselves did not think that NFTs could be applied to dementia, since the cause of dementia is due to excessive amyloid β and genetic abnormalities and has nothing to do with neuroinflammation that causes abnormalities in brain waves, until in last decade.

Future studies should include larger sample sizes and robust controls over covariates, such as disease severity, types and dosages of medications used, educational background, and lifestyle habits, including diet and regular physical exercise. Although a few research studies have used an RCT design and most used control groups, only a few attempted to control for placebo effects; therefore, it is important to use more robust methodologies for the investigations. In addition, it should be noted that research findings may not be generalized to real-world settings.

## Author contributions

MT: Writing–review and editing.
